# Association of the programmed cell death 1 (*PDCD1*) gene polymorphism with ankylosing spondylitis in the Korean population

**DOI:** 10.1186/ar2071

**Published:** 2006-10-25

**Authors:** Sang-Hoon Lee, Yeon-Ah Lee, Doo-Hyun Woo, Ran Song, Eun-Kyung Park, Mi-Hyun Ryu, Young-Hoon Kim, Kyoung-Soo Kim, Seung-Jae Hong, Myung Chul Yoo, Hyung-In Yang

**Affiliations:** 1 Department of Rheumatology, College of Medicine, Kyunghee University, 1 Hoegi-dong, Dongdaemun-gu, Seoul, 130-701, South Korea; 2 East-West Bone and Joint Research Center, Kyunghee University East-West Neo Medical Center, 149 Sangil-dong, Gangdong-gu, Seoul, 134-090, South Korea; 3 Department of Orthopedic Surgery, College of Medicine, Kyunghee University, 1 Hoegi-dong, Dongdaemun-gu, Seoul, 130-701, South Korea

## Abstract

The PD-1 (programmed death 1) molecule is a negative regulator of T cells. *PDCD1 *(programmed cell death 1) has been reported to have a genetic association in systemic lupus erythematosus and rheumatoid arthritis in Caucasians. However, there are no reports on the association between this gene and ankylosing spondylitis (AS). The present study investigated the association of the PD-1 polymorphisms and the haplotypes with AS in a Korean population sample. In a case-control association study, two single-nucleotide polymorphisms, PD-1.5 C/T and PD-1.9 T/C, were genotyped in 95 AS patients and 130 healthy controls. The T allele of the PD-1.9 polymorphism was more frequent in the Korean male population with AS than in the Korean male controls (21.0% versus 6.9%, odds ratio 1.89, 95% confidence interval 1.483 to 2.408). The frequency of the CT haplotype (PD-1.5 C/T and PD-1.9 T/C) was higher in the AS patients (19%) than the controls (5.4%) (odds ratio 1.83, 95% confidence interval 1.559 to 2.521). The PD-1 polymorphism was demonstrated in Korean AS patients. The results suggest a genetic association between the PD-1 polymorphism and susceptibility to AS.

## Introduction

Ankylosing spondylitis (AS) is an important chronic inflammatory disease with an incidence range of between 0.5% and 1.0% [1,2]. Many genetic and environmental factors have been suggested to have some role in the development of AS [3]. However, the pathogenesis of AS is still unclear. Human leukocyte antigen (HLA) B27 makes up only a small proportion of the overall risk for spondyloarthritis. Fewer than 5% of HLA-B27-positive people in the general population develop these diseases [4]. In contrast, 20% of the HLA-B27-positive relatives of AS patients are affected. Family studies have suggested that HLA-B27 contributes to about 37% of the overall genetic risk for spondyloarthritis [5-7], which suggests the involvement of other genes in the development of AS. Another new susceptible gene therefore needs to be identified.

Recently, the *PDCD1 *(programmed cell death 1) gene polymorphism was reported to be associated with systemic lupus erythematosus (SLE) [8,9], lupus nephritis [10,11] and seronegative rheumatoid arthritis (RA) [12]. It was also suggested that lupus nephritis and the seronegative RA susceptibility was associated with the PD-1.3 (position 7,146) A allele in a northern Sweden population.

The PD-1 molecule is a negative regulator of T cells [13] that belongs to the immunoglobulin receptor superfamily. It encodes a 55 kDa type 1 transmembrane inhibitory immunoreceptor and is responsible for the negative regulation of T-cell activation and peripheral tolerance [14]. Nishimura and Honjo [15] reported that PD-1 expression was observed only in activated T and B cells as well as in the early lymphoid precursors. PD-1 is actively expressed on the cell surface during the activation of T and B cells. The cytoplasmic immunoreceptor tyrosine-based inhibitory motif of PD-1 was activated by an interaction between PD-1 and its corresponding ligands, PDL-1 (B7-H1) and PDL-2 (B7-DC) [15,16], which induces the inhibitory signal to inhibit the proliferation of T and B cells to maintain peripheral tolerance [14-16].

The human gene encoding PD-1, *PDCD1*, is localized on 2q37.3, which is a susceptibility locus for SLE [10]. Allele T of a single-nucleotide polymorphism (SNP) corresponding to PD-1.5 C/T (dbSNP rs#cluster id rs2227981) was found to be associated with the development of RA (odds ratio (OR) 1.94, 95% confidence interval (CI) 1.25 to 3.01, *p *< 0.0025) but not SLE in Chinese patients living in Taiwan [17].

So far more than 30 SNPs have been identified. Among the 30 SNPs, 7 (namely PD-1.1, PD-1.2, PD-1.3, PD-1.4, PD-1.5, PD-1.6 and PD-1.9) were examined in SLE [12]. Two SNPs (PD-1.5 C/T (rs2227981) and PD-1.9 T/C/(rs2227982)) were selected because they occur in an exon, which affects protein synthesis. The change in PD-1.9 T/C causes a change in their synthesized amino acid from valine to alanine. Our study was therefore focused on determining whether PD-1.9 T/C SNP affects the development of AS.

Overall, *PDCD1 *is a strong candidate for susceptibility to autoimmune disease. This study therefore investigated whether or not *PDCD1 *is associated with AS in the Korean population.

## Materials and methods

### Patients and control subjects

Case-control genetic association studies were performed to test the association between *PDCD1 *and the development of AS. The patient group consisted of 95 Korean patients with AS who were recruited from the Kyunghee University Medical Center, Seoul, Korea. A diagnosis of AS was established by using the classification criteria reported by the American College of Rheumatology (Modified New York Criteria). At least two rheumatologists confirmed the diagnosis. The patients were diagnosed before March 2005 and samples of whole blood was extracted between March 2003 and February 2005. The other control group was made up of 130 normal individuals who underwent a health examination in Kyunghee University Medical Center. They had no previous medical history and no abnormal laboratory results. All subjects provided written informed consent. All medical records in both groups were reviewed and analysed according to age, gender, combined disease, complications and disease duration.

This study was approved by the ethics review committee of the Medical Research Institute at Kyung Hee University Medical Center, Seoul, Korea in accordance with the World Medical Association Declaration of Helsinki: Ethical Principles for Medical Research Involving Human Subjects.

### Genotyping of PD-1.5 and 1.9

**Figure 1:**
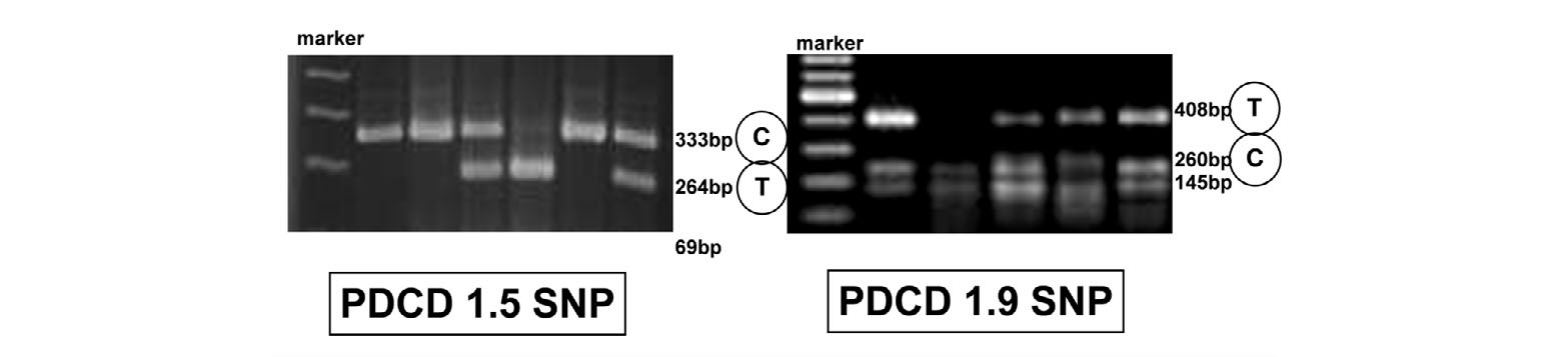
Gel electrophoresis patterns of PD-1.5 and PD-1.9. **(a) **The amplified fragments of PD-1.5 were digested with *Alu *I: the polymerase chain reaction (PCR) product size was 333 base pairs (bp), which was digested to 264 and 69 bp. If the product was digested, the allele was identified as T; if not, it was identified as C. **(b) **The amplified fragments of PD-1.9 were digested with *Bpu *10I: the PCR product size was 408 bp, which was digested to 260 and 145 bp. If the product was digested, the allele was identified as C; if not, it was identified as T.

Samples of peripheral blood were obtained from all participants after receiving informed consent. The genomic DNA was extracted from the EDTA-treated whole blood with the Wizard^® ^Genomic DNA Purification Kit (Promega, San Luis Obispo, CA, USA). PCR amplification was performed with 50 ng of the genomic DNA in a 25 μl reaction volume containing 10 pmol of sense primer in 0.5 μl, 10 pmol of antisense primer in 0.5 μl, 0.5 μl of 2.5 mmol/l dNTP (Takara, Shiga, Japan), 1 U of tag DNA polymerase (Takara), and a buffer provided by the manufacturer. The samples were subjected to 35 amplification cycles in an icycler™ Thermal Cycler (Bio-Rad, Hercules, CA, USA). The subsequent restriction-fragment-length polymorphism (RFLP) analysis was performed to determine the PD-1.5 C/T (dbSNP rs#cluster id. rs2227981) and PD-1.9 C/T (dbSNP rs#cluster id. rs2227982) SNPs using *Alu *I (New England Biolabs Inc., Beverly, MA, USA) and *Bpu *10I (New England Biolabs Inc), respectively (Table 1) [5]. Figure 1 shows the agarose gel electrophoresis results after RFLP.

### Statistical analysis

The SPSS statistical package (version 10.0) was used for statistical analysis. The data are expressed as means ± SD. The baseline differences between the groups were tested with Student's *t *test and a χ^2 ^test. All the SNP analyses, such as Hardy–Weinberg equilibrium test, linkage disequlibrium and haplotype inference, were performed with SNP Alyze (version 5.1; DYNACOM). Hardy–Weinberg equilibrium, which indicates the absence of any discrepancies between the genotype and allele frequencies, was examined and no discrepancies were noted. SNP Alyze was used to examine the allele frequency and haplotype. There was no linkage disequilibrium or haplotype inference. The ORs and 95% CIs of the allele frequencies and haplotypes were calculated in the control and disease groups. The allele frequencies were compared by using a χ^2 ^test with a 2 × 2 contingency table. A χ^2 ^test was also used to compare the haplotype frequencies. *p *≤ 0.05 was considered significant.

## Results

### Basal characteristics of patients with ankylosing spondylitis and of normal controls

Table 2 shows the basal characteristics and combined disease of the subjects. In brief, there were 83 idiopathic AS patients. The other patients had inflammatory bowel disease, reactive arthritis or psoriasis. Their age at onset, age at diagnosis and duration from initial symptoms to diagnosis were 23.1 ± 7.6 years, 25.9 ± 8.5 years and 32.1 ± 38.2 months, respectively. The percentages of patients with uveitis and peripheral joint involvement were 8.4% and 29.4%, respectively.

### PD-1.5 SNP in patients with ankylosing spondylitis and in normal controls

A total of 225 subjects (95 patients and 130 controls) were genotyped for PD-1.5 C/T SNPs. Among the 95 AS patients, 42.0%, 49.4% and 8.6% had the C/C, C/T and T/T genotypes, respectively; among the 130 normal controls these proportions were 40.8%, 39.2% and 20.0%, respectively. Among the 95 AS patients, the allele frequencies of C and T were 66.7% and 33.3%, respectively, and 60.8% and 39.2% among the normal controls. There was no significant difference between the PD-1.5 C/T SNPs and uveitis, peripheral joint involvement, total hip replacement and gender (Table 3).

### PD-1.9 SNP in patients with ankylosing spondylitis and normal controls

A total of 225 subjects (95 patients and 130 controls) were genotyped for the PD-1.9 T/T SNPs. Among the 95 AS patients, 2.5%, 37.5% and 60.5% had the T/T, C/T and C/C genotypes, respectively; among the 130 normal controls the proportions were 2.3%, 9.2% and 88.5%, respectively. There was a significant difference between the groups in C/T versus C/C but no significant difference in C/C versus T/T. Among the 95 AS patients, the allele frequencies of T and C were 21.0% and 79.0%, respectively, and 6.9% and 93.1% among the controls. There was a statistically significant difference between the groups, and the 95% CI 1.483 to 2.408, OR was 1.89 according to the χ^2 ^test (Table 3).

### Haplotype analysis

Among the 95 AS patients, 19.1%, 47.5%, 1.9% and 31.5% had the CT (PD-1.5 C/T and PD-1.9 T/C), CC, TT and TC haplotype, respectively; among the 130 normal controls the proportions were 5.4%, 55.4%, 1.5% and 37.7%, respectively. The frequency of the CT haplotype was higher in the AS patients than the controls (OR 1.83; 95% CI 1.559 to 2.521; Table 3).

## Discussion

The results show that a functionally important polymorphism in *PDCD1 *is associated with a subset of AS. A polymorphism in this gene has previously been shown to be associated with SLE [8,9] and RA [12]. These results suggest that this gene may commonly have some role in the development of autoimmune disease. *PDCD1 *has a key role in the immune response as follows.

The ligands for PD-1 have been identified as PD-L1 (B7-H1), which is expressed in all haemopoietic cells as well as in many non-haemopoietic tissues, and PD-L2 (B7-DC), which is expressed primarily on dendritic cells and macrophages [14,16,18,19]. This molecule has a role in the negative co-stimulated pathway involving the CD28 homologue PD-1 receptor [20]. PD-1 is expressed by activated, but not unstimulated, T cells, B cells and myeloid cells, which is in contrast with the predominantly T-cell-restricted expression of CD28 and CTLA-4 [21-23]. PD-1^-/- ^mice display a variety of autoimmune pathologies, including dilated cardiomyopathy (in BALB/c mice) and a lupus-like syndrome (in B6 mice). This indicates that PD-1 has a key role in the maintenance of peripheral tolerance to self-antigens, which is analogous to that of CTLA-4 [13,24]. The development of AS can therefore be affected by PD-1 expression or the function of the PD-1 molecule, and is associated with the continuous activation of immune cells such as CD4^+ ^T cells or dendritic cells. HLA B27, which is the most important genetic factor for AS, is a class I antigen and reacts with CD 8^+ ^T cells. However, most autoimmune diseases are associated with CD4^+ ^T cells. Recently, misfolding HLA B27 was reported to stimulate CD4^+ ^T cells or natural killer cells in HLA B27 transgenic rats [25]. In contrast, it was suggested that HLA B27 itself also could act as an autoantigen, because it is recognized by the T-cell receptors on CD4^+ ^T cells [26] or by its presentation by HLA class II molecules. This suggests the importance of the CD4^+ ^T-cell activation pathway and termination of its activation in AS. It is therefore possible that AS is associated with the function of the PD-1 molecule.

The PD-1 SNP has been examined in several studies but only in SLE and RA patients. Ferreiros-Vidal and colleagues [9] studied PD-1.1, PD-1.2, PD-1.3, PD-1.5, PD-1.6 and PD-1.9 SNPs in Spanish patients with SLE. They reported that the PD-1.9 minor allele was too rare and reported an incidence of less than 1.1% in 186 patients with SLE and 0% in the control group of 186 individuals. As a result, there were no more studies on PD-1.9 SNP. However, the results in the present study suggest that the PD-1.9 T allele is more frequent in a Korean population sample than in a Spanish population sample (21% in patients with AS and 6.9% in controls). The reason for this difference is that previous studies were confined to females because of the high female predominance of SLE. It should be noted that AS develops predominantly in males. Our results showed no gender difference in the frequency of PD-1.5 or PD-1.9. The PD-1.5 SNP has been studied extensively in several populations. The PD-1.5 C allele was found to be more frequent in Korean populations than in other populations (Spanish, Swedish, European and American Mexican). The data are shown in Table 4. Table 5 shows the data for another Asian group study, a Chinese population for RA [11]. In that study the control group was 33% female; in contrast, 41% of the control group in the present study were female.

The limitation of our study was that the control group was mismatched to the disease group in sex and age. With regard to sex, the polymorphism of this gene is not affected by the sex difference in our control group. With regard to age, with increasing age in the control group the possibility of being in the disease-free group increased because AS develops at a relatively young age.

The PD-1.9 T allele was found to be a susceptibility factor for AS; however, 1.5 SNP was not a factor. The PD-1.9 T allele substitutes valine for alanine 215 during PD-1 protein synthesis. However, other subgroup analyses according to gender, combined uveitis, the involvement of a peripheral joint and the existence of HLA B27 showed no significant differences. Therefore, further study will be needed to determine whether this amino acid change alters the protein structure and affects its function.

## Conclusion

*PDCD1 *was known as another genetic risk factor in RA or SLE patients. However, there was no known study on this gene with regard to AS. We have examined the association between this autoimmune-associated gene and AS and concluded that the gene is associated with susceptibility to AS, although only a small sample was used. We believe that these results are sufficient to suggest another genetic susceptibility (*PDCD1*) for AS.

## Abbreviations

AS = ankylosing spondylitis; HLA = human leukocyte antigen; OR = odds ratio; RA = rheumatoid arthritis; RFLP = restriction-fragment-length polymorphism; SLE = systemic lupus erythematosus; SNP = single-nucleotide polymorphism.

## Competing interests

The authors declare that they have no competing interests.

## Authors' contributions

SL analysed the data and drafted the manuscript. YL and SH provided subjects. DW and RS provided the subjects' data by chart review. EP performed the DNA analysis. MR, YK and KK advised on the technical protocol. MCY and HY provided subjects and contributed the conceptual design and analytical support. All authors read and approved the final manuscript.

## Figures and Tables

**Table 1:** PCR-RFLP methods, PCR primers and restriction enzymes (RE).

Polymorphism	SNP	Location	Method	RE	PCR primers
PD-1.5	7785C/T	Exon 5	PCR-RFLP	*Alu *I	5' -AGACGGAGTATGCCACCATT-3'
					5' -CACTGTGGGCATTGAGACAT-3'
PD-1.9	7625C/T	Exon 5	PCR-RFLP	*Bpu *10I	5' -GGACAGCTCAGGGTAAGCAG-3'
					5' -AGGGTCTGCAGAACACTGGT-3'

SNP, single-nucleotide polymorphism; PCR, polymerase chain reaction; RFLP, restriction-fragment-length polymorphism.

**Table 2:** Basal characteristics and clinical features of patients with AS and of normal controls.

Characteristic	Patients	Controls
Sex, M:F (%)	88:7 (92.6:7.4)	76:54 (58.5:41.5)
Age (mean ± SD)	33.4 ± 9.4	66.3 ± 5.8
Disease subgroup, *n*		
Idiopathic AS	83	
IBD	3	
Reactive arthritis	2	
Psoriatic arthritis	1	
JAS	6	
Clinical features		
Age on initial symptom, years (mean ± SD)	23.1 ± 7.6	
Disease duration, months (mean ± SD)	25.9 ± 8.5	
Delayed diagnosis, months (mean ± SD)	32.1 ± 38.2	
HLA B27^+ ^(*n *= 84) (%)	74 (88.1)	
Complications, *n *(%)		
Uveitis	8 (8.4)	
Peripheral joint involvement	28 (29.4)	
Total hip replacement	13 (13.5)	
IgA nephropathy	0 (0)	
Heart conduction abnormality	0 (0)	
Pneumonitis	0 (0)	
Cauda equina syndrome	3 (3.1)	

AS, ankylosing spondylitis; IBD, inflammatory bowel disease; JAS, juvenile ankylosing spondylitis; HLA, human leukocyte antigen.

**Table 3:** Genotyping and allele frequency and haplotype of PD-1.5 and PD-1.9 SNP in patients and controls.

SNP	Alleles	Patients (%)	Controls (%)	OR (95% CI)
PD-1.5	CC	34 (42.0)	53 (40.8)	
	CT	40 (49.4)	51 (39.2)	
	TT	7 (8.6)	26 (20.0)	
	C	108 (66.7)	158 (60.8)	
	T	54 (33.3)	102 (39.2)	
PD-1.9	TT^a^	2 (2.5)	3 (2.3)	
	CT	30 (37.5)	12 (9.2)	
	CC^b,c^	49 (60.5)	115 (88.5)	
	T^d^	34 (21.0)	18 (6.9)	1.89 (1.483–2.408)
	C	128 (79.0)	242 (93.1)	
PD-1.5/1.9 (haplotype)	CT^e^	31 (19.1)	14 (5.4)	1.83 (1.559–2.521)
	CC	77 (47.5)	144 (55.4)	
	TT	3 (1.9)	4 (1.5)	
	TC	51 (31.5)	98 (37.7)	

SNP, single-nucleotide polymorphism; OR, odds ratio; CI, confidence interval. ^a^*p *= 0.000, χ^2 ^test, in PD-1.9 SNP ; ^b^*p *= 0.638, χ^2 ^test, CC versus TT; ^c^*p *= 0.000, χ^2 ^test, CC versus CT ; ^d^*p *= 0.000, χ^2 ^test, in PD-1.9 SNP; ^e^*p *= 0.000, χ^2 ^test, in PD-1.5/1.9 haplotype analysis.

**Table 4:** Differences in PD-1.5C allele frequencies between Korean female control population and other female populations.

Population	*n*/*N *(%)
Korean	68/108 (63.0)
Spanish	540/984 (54.9)
Swedish	220/404 (54.4)
European American	84/160 (52.5)
Mexican	215/360 (59.7)

**Table 5:** Differences in PD-1.5 genotype and alleles between Korean and Chinese populations.

Characteristic	Korean controls (%)	Chinese controls (%)
Characteristic	Korean controls (%)	Chinese controls (%)
CC	53 (40.8)	333 (51.5)
CT	51 (39.2)	259 (40.0)
TT	26 (20.0)	55 (8.5)
Allele frequency		
C	158 (60.8)	925 (71.5)
T	102 (39.2)	369 (28.5)

Chinese data from [17].

## References

[B1] Johnsen K, Gran JT, Dale K, Husby G (1992). The prevalence of ankylosing spondylitis among Norwegian Samis (Lapps). J Rheumatol.

[B2] Braun J, Bollow M, Remlinger G, Eggens U, Rudwaleit M, distler A, Sipper J (1998). Prevalence of spondylarthropathies in HLA B27-positive and -negative blood donors. Arthritis Rheum.

[B3] Sieper J, Rudwaleit M (2005). How early should ankylosing spondylitis be treated with tumour necrosis factor blockers?. Ann Rheum Dis.

[B4] van der Linden SM, Valkenburg HA, de Jongh BM, Cats A (1984). The risk of developing ankylosing spondylitis in HLA-B27 positive individuals. A comparison of relatives of spondylitis patients with the general population. Arthritis Rheum.

[B5] Brown MA, Laval SH, Brophy S, Calin A (2000). Recurrence risk modeling of the genetic susceptibility to ankylosing spondylitis. Ann Rheum Dis.

[B6] Brown MA, Kennedy LG, MacGregor AJ, Darke C, Duncan E, Shatford JL, Taylor A, Calin A, Wordsworth P (1997). Susceptibility to ankylosing spondylitis in twins: the role of genes, HLA, and the environment. Arthritis Rheum.

[B7] Allen RL, Raine T, Haude A, Trowsdale J, Wilson MJ (2001). Leukocyte receptor complex-encoded immunomodulatory receptors show differing specificity for alternative HLA-B27 structures. J Immunol.

[B8] Sanghera DK, Manzi S, Bontempo F, Nestlerode C, Kamboh MI (2004). Role of an intronic polymorphism in the PDCD1 gene with the risk of sporadic systemic lupus erythematosus and the occurrence of antiphopholipid antibodies. Hum Genet.

[B9] Ferreiros-Vidal I, Gomez-Reino JJ, Barros F, Carracedo A, Carreira P, Gonzalez-Escribano F, Liz M, Martin J, Ordi J, Vicario JL (2004). Association of PDCD1 with susceptibility to systemic lupus erythematosus: evidence of population-specific effects. Arthritis Rheum.

[B10] Prokunina L, Castillejo-Lopez C, Oberg F, Gunnarsson I, Berg L, Magnusson V, Brookes AJ, Tentler D, Kristiansdottir H, Grondal G (2002). A regulatory polymorphism in PDCD1 is associated with susceptibility to systemic lupus erythematosus in humans. Nat Genet.

[B11] Prokunina L, Gunnarsson I, Sturfelt G, Truedsson L, Seligman VA, Olson JL, Seldin MF, Criswell LA, Alarcon-Riquelme ME (2004). The systemic lupus erythematosus-associated PDCD1 polymorphism PD1.3A in lupus nephritis. Arthritis Rheum.

[B12] Prokunina L, Pdyukov L, Bennet A, de Faire U, Wiman B, Prince J, Alfredsson L, Klareskog L, Alarcon-Riquelme M (2004). Association of the PD-1.3A allele of the PDCD1 gene in patients with rheumatoid arthritis negative for rheumatoid factor and the shared epitope. Arthritis Rheum.

[B13] Nishimura H, Okazaki T, Tanaka Y, Nakatani K, Hara M, Matsumori A, Sasayama S, Mizoguchi A, Hiai H, Minato N (2001). Autoimmune dilated cardiomyopathy in PD-1 receptor deficient mice. Science.

[B14] Freeman GJ, Long AJ, Iwai Y, Bourque K, Chernova T, Nishimura H, Fitz LJ, Malenkovich N, Okazaki T, Byrne MC (2000). Engagement of the PD-1 immunoinhibitory receptor by a novel B7 family member leads to negative regulation of lymphocyte activation. J Exp Med.

[B15] Nishimura H, Honjo T (2001). PD-1: an inhibitory immunoreceptor involved in peripheral tolerance. Trends Immunol.

[B16] Latchman Y, Wood CR, Chernova T, Chaudhary D, Borde M, Chernova I, Iwai Y, Long AJ, Brown JA, Nunes R (2001). PD-L2 is a second ligand for PD-1 and inhibits T cell activation. Nat Immunol.

[B17] Lin SC, Yen JH, Tsai JJ, Tsai WC, Ou TT, Liu HW, Chen CJ (2004). Association of a programmed death 1 gene polymorphism with the development of rheumatoid arthritis, but not systemic lupus erythematosus. Arthritis Rheum.

[B18] Dong H, Zhu K, Tamada K, Chen L (1999). B7-H1, a third member of the B7 family, co-stimulates T-cell proliferation and interleukin-10 secretion. Nat Med.

[B19] Tseng SY, Otsuji M, Gorski K, Huang X, Slansky JE, Pai SI, Shalabi A, Shin T, Pardoll DM, Tsuchiya H (2001). B7DC, a new dendritic cell molecule with potent costimulatory properties for T cells. J Exp Med.

[B20] Rietz C, Chen L (2004). New b7 family members with positive and negative costimulatory function. Am J Transplant.

[B21] Agata Y, Kawasaki A, Nishimura H, Ishida Y, Tsubata T, Yagita H, Honjo T (1996). Expression of the PD-1 antigen on the surface of stimulated mouse T and B lymphocytes. Int Immunol.

[B22] Vibhakar R, Juan G, Traganos F, Darzynkiewicz Z, Finger LR (1997). Activation-induced expression of human programmed death-1 gene in T-lymphocytes. Exp Cell Res.

[B23] Yamazaki T, Akiba H, Iwai H, Matsuda H, Aoki M, Tanno Y, Shin T, Tsuchiya H, Pardoll DM, Okumura K (2002). Expression of programmed death 1 ligands by murine T cells and APC. J Immunol.

[B24] Nishimura H, Nose M, Hiai H, Minato N, Honjo T (1999). Development of lupus-like autoimmune disease by disruption of the PD-1 gene encoding an ITIM motif-carrying immunoreceptor. Immunity.

[B25] Bird LA, Peh CA, Kollnberger S, Elliott T, McMichael AJ, Bowness P (2003). Lymphoblastoid cells express HLA-B27 homodimers both intracellularly and at the cell surface following endosomal recycling. Eur J Immunol.

[B26] Boyle LH, Goodall JC, Opat SS, Gaston JS (2001). The recognition of HLA-B27 by human CD4^+ ^T lymphocytes. J Immunol.

